# Characteristics and phylogenetic relationship analysis of the chloroplast genome of *Anethum foeniculum* (Apiaceae)

**DOI:** 10.1080/23802359.2022.2127338

**Published:** 2022-10-06

**Authors:** Lihua Yu, Jie Min

**Affiliations:** a Jiangxi Mental Hospital, Nanchang, China; b The Third Hospital of Nanchang, Nanchang, China

**Keywords:** *Anethum foeniculum*, Apiaceae, chloroplast genome

## Abstract

*Anethum foeniculum* is an important medicinal plant in traditional Chinese medicine. In this paper, we characterized and reported the chloroplast genome of *Anethum foeniculum.* Its sequence length is 153,618 bp, which contains a large single-copy (LSC) region of 86,651 bp, a small single-copy (SSC) region of 17,471 bp, and a pair of inverted repeats (IR) regions of 24,748 bp. The overall nucleotide composition of the chloroplast genome sequence is: A (30.8%), T (31.5%), C (19.2%), G (18.5%) and the total G + C content of 37.7%. The complete chloroplast genome sequence contains 130 genes, including 85 encoding genes, 37 transfer RNA genes and 8 ribosomal RNA genes. The phylogenetic analysis result of this study reveals that *Anethum foeniculum* is closely related to *Anethum foeniculum* (NC_029469) in the phylogenetic relationship.

*Anethum foeniculum* L. (Philip Miller 1768), commonly known as fennel, is a popular medicinal plant with various pharmacological activities in traditional Chinese medicine. This plant belonging to the carrot family (Apiaceae) has aromatic foliage, and its flavor is similar to anise. Fennel contains volatile oil substances such as anisol and anisole that have a certain sedative effect and can be consumed as a medicinal plant. In traditional medicine, it has been used to treat various ailments related to the digestive, endocrine, reproductive, and respiratory systems (Shamkant et al. 2014). In addition, fennel has the effects of dispersing cold, relieving pain, regulating the stomach, and warming the kidney (Rahimi and Ardekani 2013). In this study, we characterized the chloroplast genome of *Anethum foeniculum*, and the data provided would contribute to the elucidation of the phylogeny and evolution of *Anethum foeniculum* in the future.

Fresh leaves of *Anethum foeniculum* were collected from Nanchang City, Jiangxi Province, China (28.66 N, 115.90E). The corresponding voucher herbarium specimen (Jie Min; 23511337@qq.com) was stored in the Third Hospital of Nanchang (voucher No. THNC-06). The total genome DNA was extracted from the fresh sample of *Anethum foeniculum* using the Plant Tissues Genomic DNA Extraction Kit (TIANGEN, BJ and CN) following the protocol. The paired-end (2 × 150 bp) library was sequenced by Illumina PE150 from Sangon Biotech Co., Ltd. (Shanghai, China). After removing low-quality reads and adaptor sequences, 1.61 Gb of clean reads were obtained. Then, the chloroplast genome DNA was purified and sequenced, and FastQC version 0.11.2 (Andrews 2015) was used to control and remove the sequences. The chloroplast genome of *Anethum foeniculum* was assembled by SPAdes version 3.5.0 (Bankevich et al. 2012), which used parametric splicing sequencing data and default parameters are used in this study. The chloroplast genome sequence annotation was performed by Geneious version 10.1 (Kearse et al. 2012). All the tRNA genes were predicted using ARAGORN version 1.0 (Laslett and Canback 2004) and corrected using the NCBI Blast search. The chloroplast genome sequence of *Anethum foeniculum* was submitted to the NCBI database, with accession No. OM307067.

The complete chloroplast genome of *Anethum foeniculum* has a typical quadripartite structure. Its sequence length is 153,618 bp, which contains a large single-copy (LSC) region of 86,651 bp, a small single-copy (SSC) region of 17,471 bp, and a pair of inverted repeats (IR) regions of 24,748 bp. The overall nucleotide composition of the chloroplast genome sequence is: A (30.8%), T (31.5%), C (19.2%), G (18.5%) and the total G + C content of 37.7%. The chloroplast genome sequence contains 130 genes, including 85 encoding genes, 37 transfer RNA genes and 8 ribosomal RNA genes.

The phylogenetic relationships of *Anethum foeniculum* were reconstructed based on the chloroplast genomes of eight plant species from the same family using the maximum likelihood (ML) methods implemented on MEGA X (Kumar et al. 2018). Additionally, MEGA X was used for constructing the ML phylogenetic trees, *Arabidopsis thaliana* (NC_000932) as the outgroup. The ML phylogenetic tree was inferred with strong support and used bootstrap values of 1,000 replicates on all nodes. The ML tree was drawn and edited by MEGA X. The result of the phylogenetic analysis revealed that *Anethum foeniculum* is closely related to *Anethum foeniculum* (NC_029469) in terms of the phylogenetic relationship (Figure 1). The data provided can facilitate the future elucidation of phylogeny and evolution of *Anethum foeniculum*.

**Figure 1.**
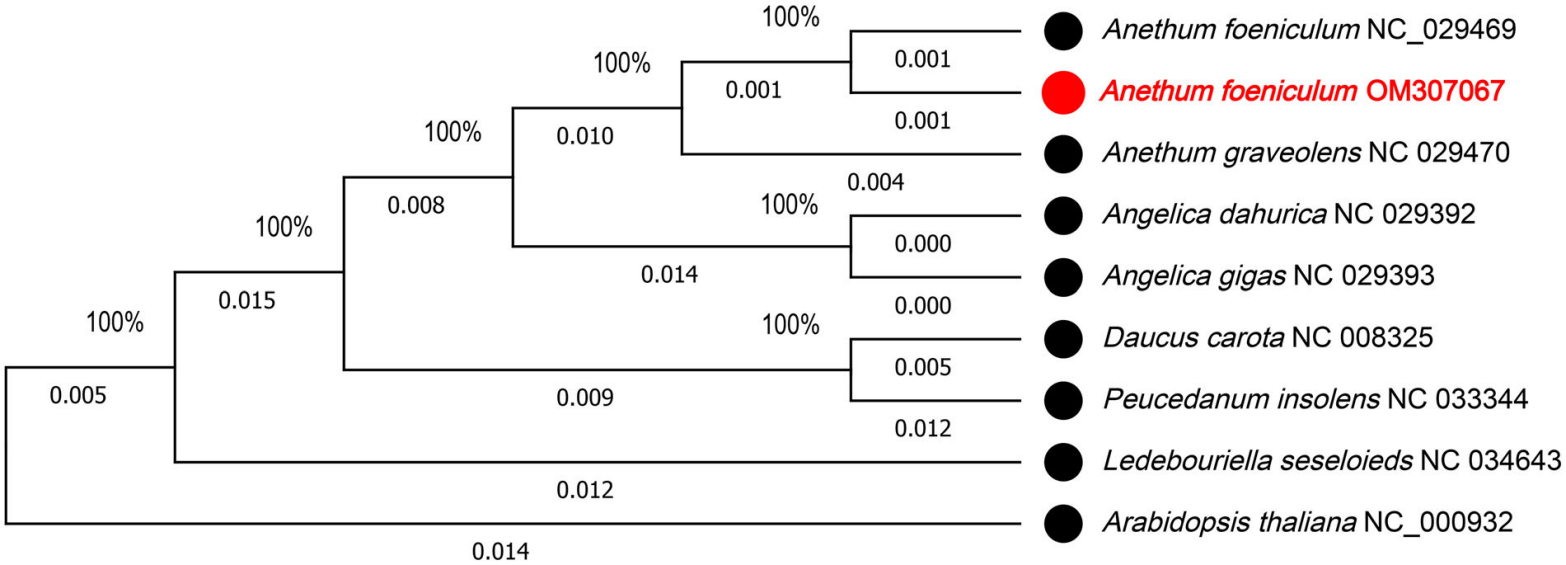
Phylogenetic relationships of *Anethum foeniculum* with eight plant species based on maximum likelihood (ML) analysis of the complete chloroplast protein-coding genes. Bootstrap support values based on 1000 replicates are shown next to the nodes. GenBank accession numbers are listed next to the species. The specie *Anethum foeniculum* is highlighted in red.

## Data Availability

The chloroplast genome sequence data supporting the findings of this study are publicly available in the GenBank of NCBI (https://www.ncbi.nlm.nih.gov/) with the accession number OM307067. The associated numbers of BioProject, SRA, and Bio-Sample are PRJNA793763, SRR17407512, and SAMN24582935, respectively.

## References

[CIT0001] Andrews S. 2015. FastQC: a quality control tool for high throughput sequence data. http://www.bioinformatics.babraham.ac.uk/projects/fastqc/.

[CIT0002] Bankevich A, Nurk S, Antipov D, Gurevich AA, Dvorkin M, Kulikov AS, Lesin VM, Nikolenko SI, Pham S, Prjibelski AD, et al. 2012. SPAdes: a new genome assembly algorithm and its applications to single-cell sequencing. J Comput Biol. 19(5):455–477. 22506599 10.1089/cmb.2012.0021 PMC3342519

[CIT0003] Kearse M, Moir R, Wilson A, Stones-Havas S, Cheung M, Sturrock S, Buxton S, Cooper A, Markowitz S, Duran C, et al. 2012. Geneious basic: an integrated and extendable desktop software platform for the organization and analysis of sequence data. Bioinformatics. 28(12):1647–1649. 22543367 10.1093/bioinformatics/bts199 PMC3371832

[CIT0004] Kumar S, Stecher G, Li M, Knyaz C, Tamura K. 2018. MEGA X: molecular evolutionary genetics analysis across computing platforms. Mol Biol Evol. 35(6):1547–1549. 29722887 10.1093/molbev/msy096 PMC5967553

[CIT0005] Laslett D, Canback B. 2004. ARAGORN, a program to detect tRNA genes and tmRNA genes in nucleotide sequences. Nucleic Acids Res. 32(1):11–16. 14704338 10.1093/nar/gkh152 PMC373265

[CIT0006] Rahimi R, Ardekani MRS. 2013. Medicinal properties of *Anethum foeniculum* Mill. in traditional Iranian medicine and modern phytotherapy. Chin J Integr Med. 19(1):73–79. 23275017 10.1007/s11655-013-1327-0

[CIT0007] Shamkant B, Vainav BV, Atmaram HB. 2014. *Anethum foeniculum* mill: a review of its botany, phytochemistry, pharmacology, contemporary application, and toxicology. Biomed Res Int. 2014:842674. 25162032 10.1155/2014/842674 PMC4137549

